# Temperature- and pH-Responsive Schizophrenic Copolymer Brush Coatings with Enhanced Temperature Response in Pure Water

**DOI:** 10.1021/acsami.2c20395

**Published:** 2023-02-03

**Authors:** Yana Shymborska, Yurij Stetsyshyn, Kamil Awsiuk, Joanna Raczkowska, Andrzej Bernasik, Natalia Janiszewska, Paweł Da̧bczyński, Andrij Kostruba, Andrzej Budkowski

**Affiliations:** † Smoluchowski Institute of Physics, Jagiellonian University, Łojasiewicza 11, 30-348 Kraków, Poland; ‡ Lviv Polytechnic National University, St. George’s Square 2, 79013 Lviv, Ukraine; § Faculty of Physics and Applied Computer Science, AGH - University of Science and Technology, al. Mickiewicza 30, 30-049 Kraków, Poland; ∥ Faculty of Food Technologies and Biotechnology, Stepan Gzhytskyi National University of Veterinary Medicine and Biotechnologies Lviv, Pekarska 50, 79000, Lviv, Ukraine

**Keywords:** polymer brushes, ‘schizophrenic’ copolymers, thermoresponsive polymer, wettability, transfer radical polymerization, LCST, water structure

## Abstract

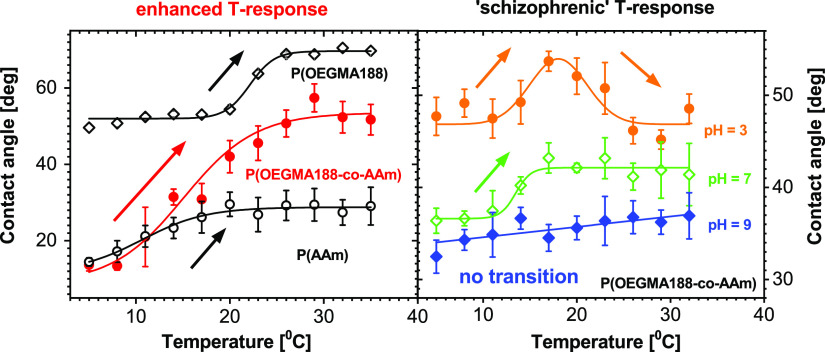

Novel brush coatings were fabricated with glass surface-grafted chains copolymerized using surface-initiated atom transfer radical polymerization (SI-ATRP) from 2-(2-methoxyethoxy)ethyl methacrylate (OEGMA188) and acrylamide (AAm), taken in different proportions. P(OEGMA188-*co*-AAm) brushes with AAm mole fraction >44% (determined with XPS and TOF-SIMS spectroscopy) and nearly constant with the depth copolymer composition (TOF-SIMS profiling) exhibit unusual temperature-induced transformations: The contact angle of water droplets on P(OEGMA188-*co*-AAm) coatings increases by ∼45° with temperature, compared to 17–18° for POEGMA188 and PAAm. The thickness of coatings immersed in water and the morphology of coatings imaged in air show a temperature response for POEGMA188 (using reflectance spectroscopy and AFM, respectively), but this response is weak for P(OEGMA188-*co*-AAm) and absent for PAAm. This suggests mechanisms more complex than a simple transition between hydrated loose coils and hydrophobic collapsed chains. For POEGMA188, the hydrogen bonds between the ether oxygens of poly(ethylene glycol) and water hydrogens are formed below the transition temperature *T*_c_ and disrupted above *T*_c_ when polymer–polymer interactions are favored. Different hydrogen bond structures of PAAm include free amide groups, *cis-trans*-multimers, and *trans*-multimers of amide groups. Here, hydrogen bonds between free amide groups and water dominate at *T* < *T*_c_ but structures favored at *T* > *T*_c_, such as *cis-trans*-multimers and *trans*-multimers of amide groups, can still be hydrated. The enhanced temperature-dependent response of wettability for P(OEGMA188-*co*-AAm) with a high mole fraction of AAm suggests the formation at *T*_c_ of more hydrophobic structures, realized by hydrogen bonding between the ether oxygens of OEGMA188 and the amide fragments of AAm, where water molecules are caged. Furthermore, P(OEGMA188-*co*-AAm) coatings immersed in pH buffer solutions exhibit a ‘schizophrenic’ behavior in wettability, with transitions that mimic LCST and UCST for pH = 3, LCST for pH = 5 and 7, and any transition blocked for pH = 9.

## 1 Introduction

Due to a critical shortage of tissue and organ donors,^1,2^ Langer and Vacanti have proposed an alternative strategy for their repair and regeneration.^3^ It is based on the manipulation of cell and tissue engineering using polymer coatings with a temperature response,^4,5^ driven in aqueous media by a lower critical solution temperature (LCST) of polymers, such as poly(*N*-isopropylacrylamide) (P*N*IPAM).^6^ By reducing the temperature around LCST, cells cultured on polymer coatings can be harvested as an intact contiguous cell sheet and transplanted into host tissue.^7^ The most promising coatings have been fabricated using polymer brushes,^8,9^ i.e., polymer chains tethered by one end to a solid surface.^10,11^ Brush coatings based on P*N*IPAM or poly(oligo(ethylene glycol) methacrylate) (POEGMA) are often used in biomedical applications. Poly(oligo(ethylene glycol) methacrylate)s are a large group of polymers that have a similar chemical structure where the carboxylic groups in methacrylic units are esterified with short chains of ethylene oxide.^12^ Their classification is often based on the molecular mass of OEGMA units, which depend on the molecular mass of short chains of ethylene oxide in the structure of the OEGMA. The most often used are polymers with a molecular mass of monomer units equal to 188 – POEGMA188 (other names poly(di(ethylene glycol)methyl ether methacrylate) or poly(2-(2-methoxyethoxy)ethyl methacrylate)). POEGMAs are biocompatible, uncharged, water-soluble, nontoxic, nonimmunogenic, and therefore the most commonly applied synthetic polymers in the biomedical field.^12^

The potential of polymer brush coatings in ‘smart’ tissue engineering is limited by several barriers, such as inappropriate wettability, weak wettability response to temperature, transition far from physiological temperature, unexpected behavior in pH buffer solutions, insufficient biocompatibility and cell specific toxicity, and difficulties with protein desorption and cell detachment. For example, as mentioned previously, P*N*IPAM-based coatings are already commercially available and are used in human patients around the world. They are not cytotoxic to the four cell types evaluated in a direct contact test (endothelial, epithelial, smooth muscle, and fibroblasts), but cell sensitivity to P*N*IPAM varies depending on the cell type.^13^ Additionally, *N*-isopropyl acrylamide, which can detach from P*N*IPAM macromolecules as a result of degradation, exhibits toxicity even at very low concentrations.^13,14^

One of the main strategies to eliminate these disadvantages is the fabrication of brush coatings with a homopolymer replaced by a copolymer.^15,16^ To this end, copolymers with monomeric units of distinct properties are grafted to solid substrates. For example, poly(*N*IPAM-*co*-2-carboxyisopropylacrylamide) brush coatings can be easily functionalized with biomolecules.^17^ Cationic brushes of poly(*N*IPAM-*co*-*N,N*-dimethylaminopropylacrylamide-*co*-*N*-*tert*-butylacrylamide) and poly(*N*IPAM-co-3-acrylamidopropyl trimethylammonium chloride-*co*-*N*-*tert*-butylacrylamide) can be used for thermally modulated purification of the human bone marrow mesenchymal stem.^18^ In turn, copolymer brushes based on a wide family of OEGMAs include those synthesized using 2-(2-methoxyethoxy)ethyl methacrylate) (OEGMA188) and hydroxyl-terminated OEGMA360, which can be modified with the RGD peptide or the antibacterial peptide, magainin.^19^ Copolymer brush coatings synthesized from poly(4-vinylpyridine-*co*-OEGMA246) exhibit a temperature-controlled three-stage switching of wetting and adsorption of bovine serum albumin.^15,16^ In a similar work,^20^ a series of copolymer brushes with temperature-switchable hydrophilic–hydrophobic balance, based on OEGMA188 and 4-vinyl pyridine, were successfully synthesized and protein adsorption was studied using isothermal titration calorimetry, demonstrating that the presence of 4-vinyl pyridine fragments in polymer brushes favored protein adsorption, below and above LCST. Finally, poly(*N*IPAM-*co*-acrylamide) brush coatings [P(*N*IPAM-*co*-AAm)] promote cell adhesion and detachment by a hydrophobic to hydrophilic transition triggered by temperature.^21−23^

On the other hand, there is great interest in ‘schizophrenic’ copolymers that show contradictory properties switched by external conditions. The phrase was coined by Armes et al.^24^ for *block* copolymers that self-assemble in dilute aqueous solutions into micelles and inverted micelles. Transformations between both micelle structures are induced by copolymer blocks tuned between hydrophilic and hydrophobic by response parameters, such as temperature, pH, or ionic strength.^25−28^ In the case of temperature-induced ‘schizophrenic’ transformations, block copolymers can exhibit transitions similar to both LCST and an upper critical solution temperature (UCST).^25,26^ Recently, ‘schizophrenic’ behavior was also demonstrated for alternating copolymers^29^ and gradient copolymers.^30^ So far, the ‘schizophrenic’ character has been reported for copolymers self-assembled into aggregates, but never for (*non-block*) copolymer brushes grafted onto solid surfaces.

Impelled by extensive research on ‘smart’ switchable copolymer materials, here, we report the fabrication and complete characterization of novel temperature- and pH-responsive copolymer brush coatings, based on poly(di(ethylene glycol)methyl ether methacrylate)-*co*-acrylamide) (P(OEGMA188-*co*-AAm)), with enhanced temperature-induced response in wettability modified to ‘schizophrenic’ by immersion in pH buffer solutions. This study shows that the contact angle (CA) of the water droplets, determined for the novel copolymer brush coatings as a function of temperature, mimics the LCST behavior but increases more dramatically than for reference homopolymer brushes (45 vs 17–18°). The wide range and well-expressed thermal response in wettability exhibited by the fabricated coatings might remove at least some disadvantages limiting applications of polymer brushes. The work also demonstrates that copolymer coatings immersed in pH buffer solutions exhibit ‘schizophrenic’ behavior in wettability, with transitions that mimic LCST and UCST for pH = 3, LCST for pH = 5 and 7, and any transition blocked for pH = 9. In this study, a series of novel brush coatings were fabricated using atom transfer radical polymerization, with glass surface-grafted chains copolymerized from OEGMA188 and AAm, taken in different proportions. The composition of grafted copolymers was determined with X-ray photoelectron spectroscopy (XPS), and it correlates well with the results of time-of-flight secondary ion mass spectrometry (ToF-SIMS). The thermal response was studied as a function of polymer brush composition for the wettability of coatings (CA), the thickness of coatings immersed in water (examined with white light reflectance spectroscopy, WLRS), and the morphology imaged in air (with atomic force microscopy, AFM). The recorded data suggest T-response mechanisms more complex than a simple transition between hydrated loose coils and hydrophobic collapsed chains. Finally, wettability and morphology were examined for copolymer coatings immersed in pH buffer solutions with different pH values.

## 2 Experimental Section

### 2.1 Materials

2-Bromoisobutyryl bromide (BIBB), (3-aminopropyl)triethoxysilane (APTES), CuBr_2_, 2,2′-dipyridyl (bpy), triethylamine (Et_3_N), sodium l-ascorbate, di(ethylene glycol)methyl ether methacrylate) (OEGMA188), acrylamide (AAm), and solvents were supplied by Sigma-Aldrich. Acid and neutral buffer solutions were prepared by mixing 0.2 M Na_2_HPO_4_ with citric acid with different ratios: pH = 3 (4.11 mL 0.2 M Na_2_HPO_4_ and 15.89 mL 0.1 M citric acid); pH = 5 (10.30 mL 0.2 M Na_2_HPO_4_ and 9.70 mL 0.1 M citric acid); pH = 7 (16.47 mL 0.2 M Na_2_HPO_4_ and 3.53 mL 0.1 M citric acid), while the alkaline one with pH = 9.2 was prepared by mixing 2.36 mL of H_2_O and 7.64 mL of 5 g/dL sodium borate (Na_2_B_4_O_7_·10H_2_O). All reagents used for buffer preparation were purchased from Sigma-Aldrich.

### 2.2 Preparation of the Samples

#### 2.2.1 Modification of Glass Surfaces with the ATRP Initiator

Glass plates (20 × 20 mm) were placed in a vacuum desiccator or vacuum oven with a vial containing 10 drops of APTES (Supporting Information, step (2) in Scheme S1). The chamber was then pumped down to <1 mbar, isolated from the pump, and left under vacuum for 30 min. The substrates were then annealed at 110 °C in air at atmospheric pressure for 30 min.

After annealing, the substrates can be directly reacted with 2-bromoisobutyryl bromide (step (3) in Scheme S1). For this, 10 mL of anhydrous tetrahydrofuran was mixed with 2-bromoisobutyryl bromide (0.26 mL, 2.10 mmol) and anhydrous triethylamine (0.30 mL, 2.10 mmol) and added to an amino-functionalized substrate.

#### 2.2.2 Polymerization of POEGMA188 Brushes (Surface-Initiated Activators Regenerated by Electron Transfer Atom Transfer Radical Polymerization (SI-ARGET ATRP))

The procedure of modification is sketched in the Supporting Information**.**

Glass plates with grafted ATRP were placed in test tubes, deoxygenated by nitrogen purging or vacuum/nitrogen cycling. Methanol, water, OEGMA, AAm, or their combinations in the concentrations (*C*) in the moles and grams presented in Table 1 were mixed in a round-bottom flask sealed with a septum and deoxygenated by bubbling through nitrogen for 10–15 min. Then, CuBr_2_, 2,2′-dipyridyl, and sodium l-ascorbate were added, and the headspace was purged with nitrogen. The mixture was stirred to dissolve the solids. Subsequently, the solution was syringed over the substrates in the deoxygenated tubes or simply poured over the substrates in a screw-top jar, which was then resealed. The samples were allowed to polymerize at room temperature. After 12 h of polymerization, the samples were removed and washed with ethanol and water.

**Table 1:** Composition of Reaction Mixtures Used for Copolymer Synthesis^a^

type			POEGMA	P(OEGMA-*co*-AAm)1	P(OEGMA-*co*-AAm)2	P(OEGMA-*co*-AAm)3	P(OEGMA-*co*-AAm)4	P(OEGMA-*co*-AAm)5	PAAm
monomers	OEGMA188	*C* [mmol]	60	54	48	30	12	6	0
		*C* [g]	11.28	10.15	9.02	5.64	2.26	1.13	0
	AAm	*C* [mmol]	0	6	12	30	48	54	60
		*C* [g]	0	0.43	0.85	2.13	3.41	3.83	4.26
catalysts	copper(II) bromide	*C* [mmol]	0.033	0.033	0.033	0.033	0.033	0.033	0.033
		*C* [g]	7.4	7.4	7.4	7.4	7.4	7.4	7.4
	2,2′-dipyridyl	*C* [mmol]	0.33	0.33	0.33	0.33	0.33	0.33	0.33
		*C* [g]	51.5	51.5	51.5	51.5	51.5	51.5	51.5
	sodium l-ascorbate	*C* [mmol]	0.33	0.33	0.33	0.33	0.33	0.33	0.33
		*C* [g]	65.3	65.3	65.3	65.3	65.3	65.3	65.3
solvents	methanol	Volume [ml]	16	16	16	14	10	10	10
	water	Volume [ml]	4	4	4	6	10	10	10

a Numbers **n** denote the synthesized P(OEGMA-*co*-AAm)**n** batches.

#### 2.2.3 Immersion in Buffer Solutions

The sample was immersed for 15 min in a buffer solution with the required pH, then carefully dried, and finally placed in the temperature-controlled chamber of the EasyDrop instrument. Before exposure to buffer that led to measurement at different pH values, the sample was immersed in deionized water for 30 min and dried under stream of nitrogen.

### 2.3 Characterization of Coatings

#### 2.3.1 X-ray Photoelectron Spectroscopy (XPS)

The X-ray photoelectron spectroscopy measurements were performed with a PHI VersaProbe II apparatus. The samples were irradiated with a focused monochromatic Al Kα (*E* = 1486.6 eV) X-ray beam with a diameter of 100 μm, and the beam was rastered over an area of 400 × 400 μm^2^. The pass energy of the analyzer was set to 46.95 eV, and double neutralization with electrons and low energy monoatomic Ar^+^ ions was used to avoid charging effects. Spectra were referenced to the neutral (C–C) carbon C 1s peak, at a binding energy of 284.80 eV.

#### 2.3.2 Time-of-Flight-Secondary Ion Mass Spectrometry (TOF-SIMS)

To examine the surface chemistry, TOF-SIMS was performed using the TOF.SIMS 5 instrument (ION-TOF GmbH), equipped with a 30 keV bismuth liquid metal ion gun. Bi_3_ clusters were used as primary ions with an ion dose density lower than 10^12^ ions/cm^2^ to ensure static mode conditions. A pulsed low-energy electron flood gun was used for charge compensation. For each sample, high mass resolution spectra were acquired from two different and nonoverlapping spots (100 μm × 100 μm area). Mass calibration was performed with H^+^, H_2_^+^, CH^+^, C_2_H_2_^+^, and C_4_H_5_^+^ peaks. The complex multiple TOF-SIMS spectra were examined with principal component analysis (PCA) to enhance the detection of subtle differences in surface chemistry between different samples.^31−34^ To perform PCA, the PLS Toolbox (Eigenvector Research, Manson, WA, USA) was applied to MATLAB (MathWorks, Inc., Natick, MA, USA). In addition to surface spectroscopy mode that applies a Bi_3_^+^ (30 keV) ion beam, the dual beam depth profiling mode, using a sputtering C_60_^+^ (10 keV) ion beam and analyzing the Bi_3_^+^ (30 keV) ion beam, was used to examine the synthesized brush coatings. Depth profiles were collected in non-interlaced mode in sequence: 1 frame of analysis, 1 frame of sputtering, and 1 s pause.

#### 2.3.3 White Light Reflectance Spectrometry (WLRS)

WLRS measurements were performed using an FR-pRo tool by ThetaMetrisis SA (Athens, Greece) combined with a liquid cell (FR-Microfluidic kit). The FR-pRo tool consists of three main elements: a broad-band UV–VIS 250–700 nm light source, a PC-driven spectrometer (Ocean Optics Maya2000 pRo), and a fiber optic reflection probe (6 + 1 fibers) from Ocean Optics operating in the corresponding spectral range. The white light emitted from the light source is guided to the reflection probe and is incident vertically onto the sample under investigation. The reflection probe is kept at a distance of 5 mm from the sample and has an active spot size of ∼1 mm in diameter. Both the sample and the probe are submerged underwater. The sample (brush coating) is placed in the docking station with a fluidic compartment (20 μL volume), built-in temperature controller (FR-Hot/Cool unit working with 0.1 °C precision), and microfluidic syringe pomp for liquid circulation with programmable speed. Measurements were made in at least three different points in each sample to monitor spatial uniformity. The light beam interacts with the sample and is collected back by the probe, while reflectance is continuously recorded and analyzed by the embedded spectrometer. ThetaMetrisis FR-Monitor software was used to record the spectra and fit the experimental data (after normalization with respect to the dark and reference spectra with known reflectivity) for the calculation of the polymer coating thickness. The fitting of the experimental spectrum was performed using the Levenberg–Marquardt algorithm, considering the refractive indices *n*(λ) of all layers of a multilayer stack of water/polymer coating/substrate, fitted to a Cauchy model of three parameters.^35,36^ For each polymer brush coating with a specific composition and structure, an optimal fitting model was chosen for the software to determine the thermal response between 5 and 35 °C. Therefore, to compare for different brush coatings the temperature variations of their thicknesses, the latter are not presented as absolute values. Instead, the values of the relative thickness of the brush coating are used in relation to those at 35 °C.

#### 2.3.4 Water Contact Angle Measurements (CA)

Static contact angle experiments were performed using the sessile drop technique using a Kruss EasyDrop instrument (DSA15) with the Peltier temperature-controlled chamber. Measurements were carried out at temperatures ranging from 5 to 35 °C to determine the thermal response of grafted brush coatings. The temperature was measured by a thermocouple (located in the docking station) in contact with the sample surface. Contact angles were expressed as the average of ten measurements at different spots.

#### 2.3.5 Atomic Force Microscopy (AFM)

Topographic images were recorded in randomly chosen regions of the sample surface. Measurements were carried out in air using the commercially available Agilent 5500 system (Keysight) equipped with a temperature controlled sample plate. They were performed in non-contact mode with non-coated super sharp silicon probes. For each temperature, at least three images were analyzed, and the size of the images was 1 × 1 μm^2^.

#### 2.3.6 Ellipsometry

The thickness of the polymer brushes was measured using a spectroscopic ellipsometer (SENTECH SpectraRey/3) equipped with a microspot. The ellipsometric angles Ψ and Δ were determined for wavelengths λ in a spectral range between 320 and 800 nm. Measurements were taken at angles of incidence and detection of 56° and 70°. Polymer layers were modeled as a Cauchy layer of thickness *d* with a refractive index *n*(*λ*) of the form: *n*(λ) = *n*_0_ + *n*_1_λ^–2^ + *n*_2_λ^–4^.

Layer thickness *d* and the set of parameters *n*_0_, *n*_1_, and *n*_2_ were numerically varied to achieve the best agreement between the model and the measured values Δ(λ) and Ψ(λ). Refractive indices of glass substrates were measured independently within the same spectral range and were accounted for in the model.^37^

## 3 Results and Discussion

In the presented work, we synthesized for the first time and complexly characterized a series of temperature-responsive copolymer brush coatings based on OEGMA188 and AAm monomers. Copolymer brush coatings were synthesized using the standard scheme (see Scheme S1 in the Supporting Information) where glass surfaces were initially functionalized by (3-aminopropyl)triethoxysilane, then with the ATRP initiator, and finally with homopolymer or copolymer brushes using different ratios of the monomers in a reactive mixture. It should be noted that toxicity in ATRP products can arise from contamination of the residual catalyst or other reaction components.^38^ In the system presented here, the samples were washed multiple times to clean the grafted brush coatings from the residual amount of the Cu catalyst. The synthesis of the grafted brush coatings was confirmed by XPS, ToF-SIMS, and ellipsometry and described in Subsection 3.1. In turn, the impact of temperature on wettability (evaluated with measurements of wetting contact angles), coating morphology (imaged in air with AFM), and the thickness of coatings immersed in water (examined with WLRS) is analyzed in Subsection 3.2 as a function of the coating composition. Finally, the pH-responsive properties of POEGMA188, P(OEGMA188-*co*-AAm), and PAAm grafted brush coatings, especially the ‘schizophrenic’ behavior of P(OEGMA188-*co*-AAm)**5** with a critically high content of AAm mers, are described in Subsection 3.3.

### 3.1 Characterization of Surface-Grafted P(OEGMA188-*co*-AAm) Brush Coatings (XPS, ToF-SIMS, Ellipsometry)

**Figure 1:**
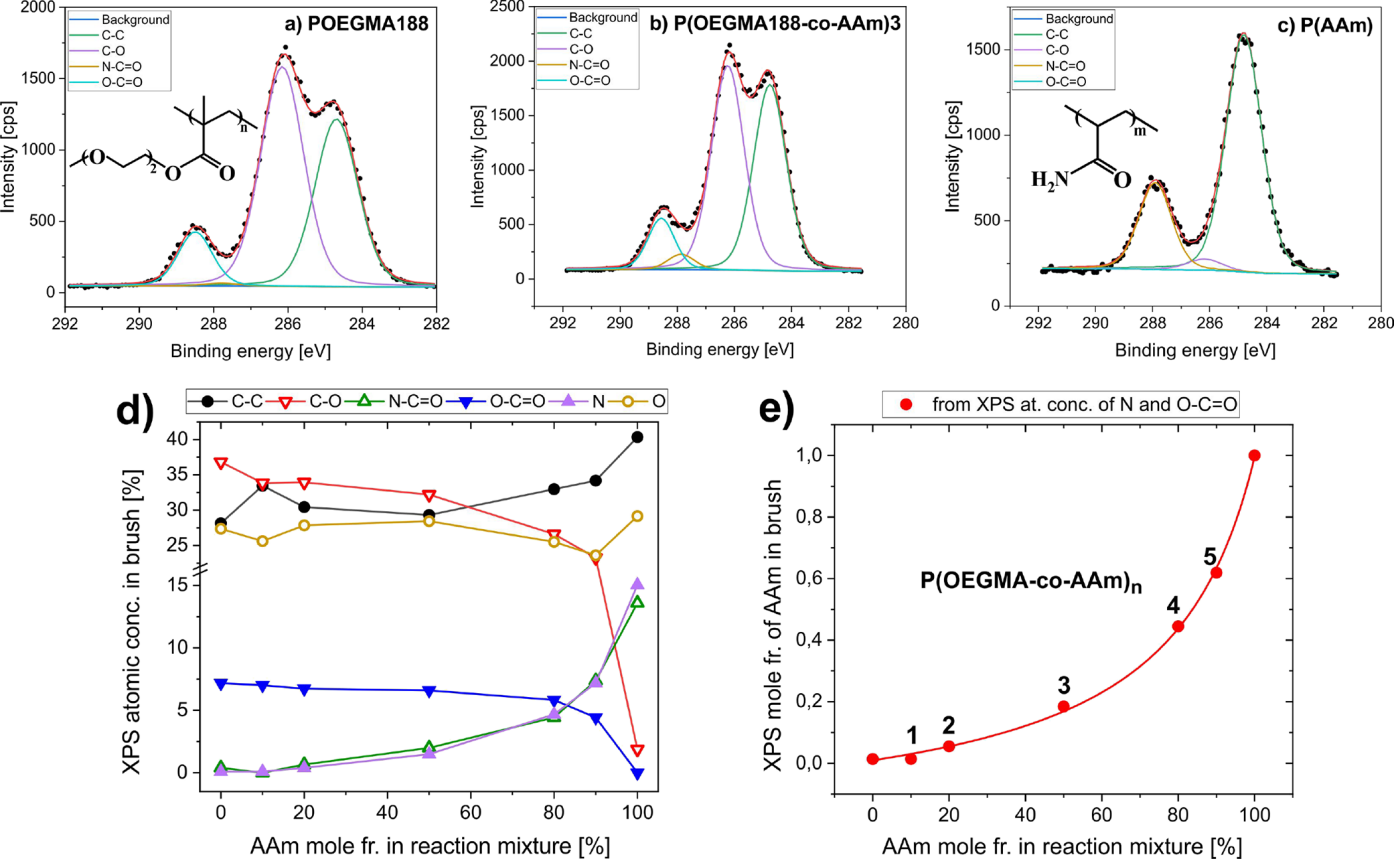
XPS C1s core-level spectra of (a) POEGMA188, (c) PAAm, and (b) P(OEGMA188-*co*-AAm) coatings (represented by the batch P(OEGMA-*co*-AAm)**3**). XPS composition of P(OEGMA188-*co*-AAm) copolymer brushes versus composition of reaction mixtures used for brush synthesis: (d) XPS atomic concentrations of elements and elements in functional groups characteristic for OEGMA188 and AAm and (e) XPS mole fraction of AAm in synthesized brushes. Numbers **n** denote the synthesized P(OEGMA188-*co*-AAm)**n** batches.

The fabricated copolymer brushes and their composition were examined using XPS and ToF-SIMS methods. First, XPS data were collected for photoelectrons emitted by the elements characteristic for the P(OEGMA188-*co*-AAM) coatings, such as carbon (C1s) and oxygen (O1s), specific for both OEGMA188 and AAm mers, as well as nitrogen (N1s), unique for AAm. In particular, the C1s core-level spectra (Figure 1) can be resolved into a few contributions, including those characteristic for either OEGMA188 or AAm. The C1s spectrum of POEGMA188 brush (Figure 1a) consists of three peaks, corresponding to neutral carbon C–C (green line, 284.8 eV), and two carbons with electron-efficient environments characteristic for OEGMA188 (with the molecular formula shown in the inset of Figure 1a): C–O (violet line, 286.3 eV) and O–C=O (cyan line, 288.6 eV) bonds. In turn, the PAAm brush (Figure 1c) exhibits, in addition to the peak of the neutral carbon C–C (284.8 eV), also a contribution from the N–C=O (brown line, 287.9 eV) bond, specific for AAm (see the inset of Figure 1c). Additionally, a significantly lower peak at 286.3 eV, corresponding to the C–O bond is observed. Finally, the spectra recorded for P(OEGMA188-*co*-AAm) coatings (represented with Figure 1b for the batch P(OEGMA188-*co*-AAm)**3**) are composed of four peaks, corresponding C–C, C–O, N–C=O, and O–C=O bonds, as expected for a copolymer.

Based on XPS data, the atomic concentrations of elements and elements in functional groups, characteristic for OEGMA188 and Aam in the copolymer brushes, were determined (Figure 1d). A monotonic increase of atomic concentrations distinctive for AAm (of nitrogen and carbon in the N–C=O bond, triangle-up symbols) is observed with increasing AAm mole fraction in the reaction mixture used for polymerization. It is accompanied by a monotonic decrease of the atomic concentrations characteristic for OEGMA188 (of carbon in C–O and O–C=O bonds, triangle-down symbols). These changes in atomic concentrations are more rapid for higher AAm concentrations in the reaction mixture, indicating a more substantial increase in the content of the AAm units in the fabricated copolymer brush coatings. To determine the mole fraction of AAm in the copolymer brushes (Figure 1e), the atomic concentrations of nitrogen and carbon in O–C=O bonds were used since the other two characteristic concentrations do not reach a zero value for pure polymer brushes (i.e., carbon in the N–C=O bond for POEGMA188 and carbon in the C-O bond for PAAm). The evaluated mole fraction of AAm in the synthesized brushes (Figure 1e) increases monotonically, slowly, and then more rapidly with AAm concentration in the reaction mixture, as noted above.

**Figure 2:**
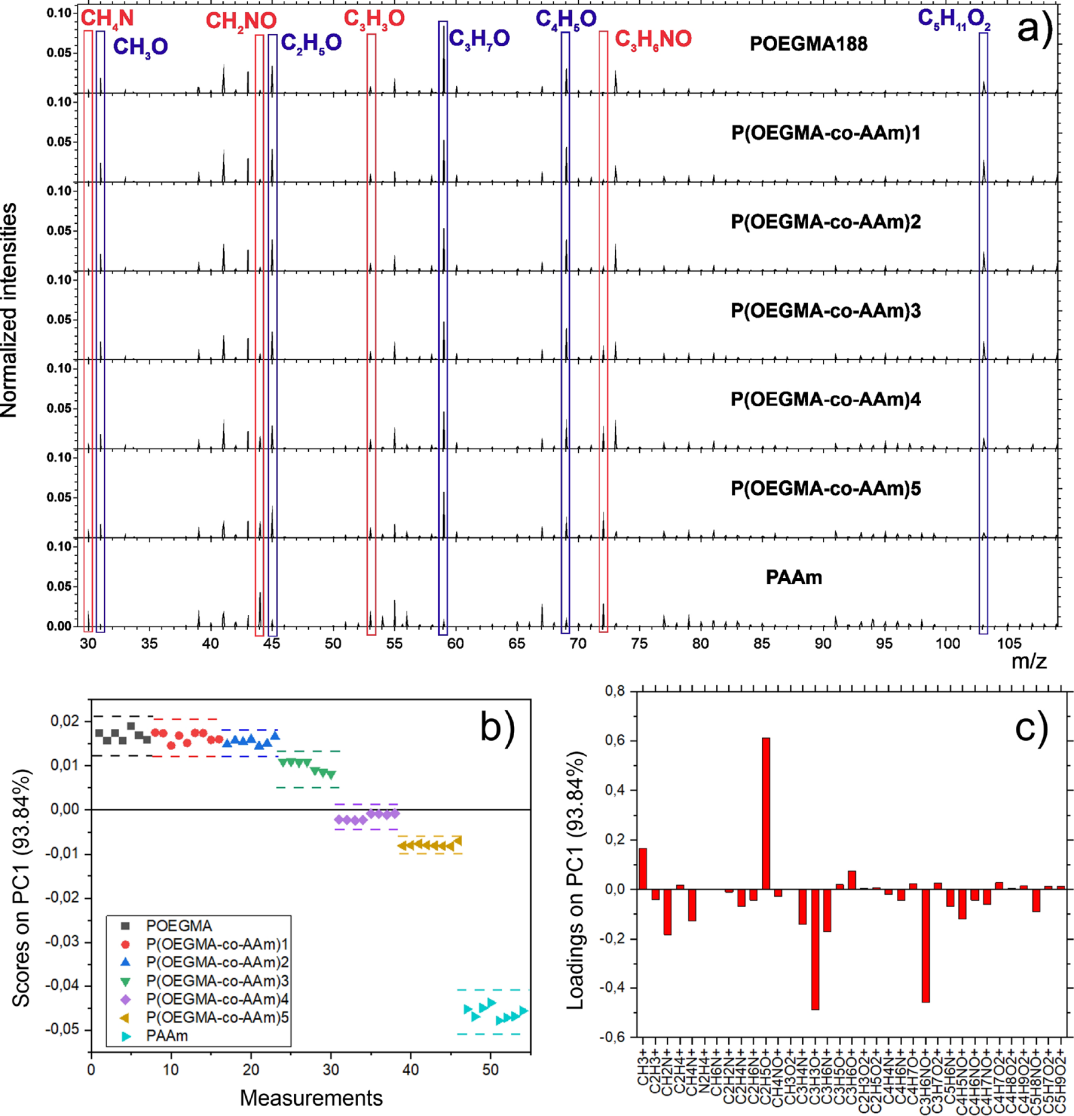
(a) Positive ToF-SIMS spectra collected from POEGMA188, PAAm, and P(OEGMA188-*co*-AAm) copolymer brush coatings. Ions characteristic for OEGMA188 (CH_3_O^+^, C_2_H_5_O^+^, C_3_H_7_O^+^, C_4_H_5_O^+^, and C_5_H_11_O_2_^+^) and AAm (CH_4_N^+^, CH_2_NO^+^, C_3_H_3_O^+^, and C_3_H_6_NO^+^) are marked in blue and red, respectively. Signal intensities are normalized by the total ion intensity *I*_tot_ of each spectrum. (b) PCA scores plot of the P(OEGMA188-*co*-AAm) copolymer as well as POEGMA188 and PAAm polymer brush coatings. The dashed lines represent the 95% confidence limits for each group of data points. (c) Corresponding loading plot that relates PC1 with ToF-SIMS signals.

Successful synthesis of P(OEGMA188-*co*-AAm) copolymer brushes with varied content of AAm monomeric units was confirmed using the ToF-SIMS method. Analysis of ToF-SIMS spectra (Figure 2a) reveals a series of signals CH_3_O^+^, C_2_H_5_O^+^, C_3_H_7_O^+^, C_4_H_5_O^+^, and C_5_H_11_O_2_ (marked in blue) that are characteristic for OEGMA188. The normalized intensities of these peaks decrease with increasing batch number **n** of synthesized copolymer P(OEGMA188-*co*-AAm)**n**, confirming lower fraction of OEGMA188 units for higher content of AAm in the reaction mixture (cf. Figure 2b). In turn, there are several peaks specific for AAm units in copolymer brush coatings, CH_4_N^+^, CH_2_NO^+^, C_3_H_3_O^+^, and C_3_H_6_NO^+^ (marked in red in Figure 2a), with normalized intensities clearly increasing with higher AAm content during synthesis (cf. Figure 2b).

To enhance ToF-SIMS sensitivity to subtle variations in the chemistry of synthesized polymer and copolymer brushes, the normalized intensities of 38 chosen signals (listed in Figure 2c) of 56 spectra were simultaneously inspected with a multivariate principal component analysis. The directions of major uncorrelated variations within the ToF-SIMS data set, so-called principal components PCs, are determined within the PCA analysis. The first principal component (PC1) already captures most of the total variance in the data set (93.84%). The relation of PC1 with the original mass signals is presented in Figure 2c. It is clear that the positive loadings on PC1 are dominated by the C_2_H_5_O^+^ signal characteristic for OEGMA188. In turn, negative PC1 loadings are mainly contributed by C_3_H_3_O^+^ and nitrogen containing fragments originating from AAm. Hence, PC1 resolves between the brush composition rich in OEGMA188 and in AAm. Indeed, the scores on PC1 (Figure 2b) indicate that PC1 is spanned by the data of pure polymer brushes, POEGMA188 (positive scores) and PAAm (negative scores), while the data of P(OEGMA188-*co*-AAm)**n** copolymer brushes are located between these data groups with the PC1 scores decreasing with batch number **n**, confirming higher AAm content for higher AAm concentration in the reaction mixture (cf. Figure 1e).

**Figure 3:**
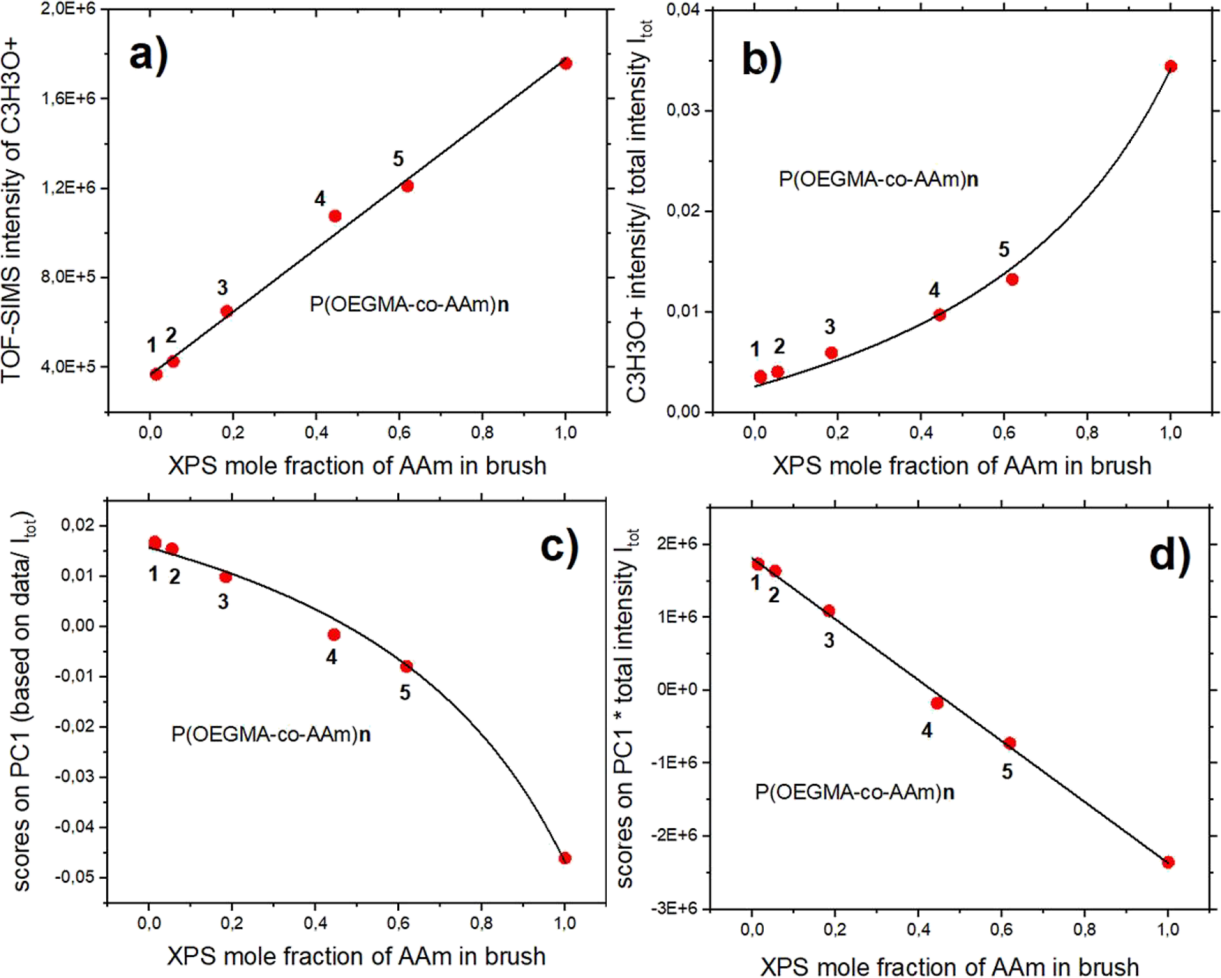
Comparison of ToF-SIMS and XPS data for the synthesized P(OEGMA188-*co*-AAm) brush coatings. The ToF-SIMS results, quantified using the univariate calibration (a, b) and the multivariate PCA approach (c, d), are plotted as a function of the XPS mole fraction of AAm. (a) Linear relation of the absolute intensity of the C_3_H_3_O^+^ signal characteristic for AAm. (b) Slightly nonlinear dependence of the C_3_H_3_O^+^ signal intensity normalized by total ion intensity *I*_tot_, following a relation described as the ratio of two linear expressions. (c) Dependence of the scores on PC1, based on the analysis of normalized ToF-SIMS intensities, and, therefore, following the relation used in panel (b). (d) Linear relation for the scores on PC1 multiplied by the total ion intensity *I*_tot_. Numbers **n** denote the synthesized P(OEGMA188-*co*-AAm)**n** batches.

It is of interest to compare the quantitative results of XPS, which exhibits a low detection limit, with the highly molecular sensitive information of ToF-SIMS, which does not possess intrinsic callibration.^39^ For the synthesized P(OEGMA188-*co*-AAm) brush coatings, the obtained ToF-SIMS data can be quantified using the univariate calibration (Figure 3a,b) and the multivariate PCA approach (Figure 3c,d) to be directly related to the mole fraction of AAm determined by XPS (Figure 1e). The linear relationship between the absolute ToF-SIMS intensity of the ion fragment and the XPS composition of the copolymer brush is observed for the C_3_H_3_O^+^ signal characteristic for AAm (Figure 3a), indicating (in contrast to some other signals) a negligible matrix effect on the ion formation.^39^ Commonly, absolute intensities of mass signals are normalized by the total ion intensity *I*_tot_, which also depends linearly on the composition. Therefore, the normalized intensity varies slightly nonlinearly with the copolymer composition (Figure 3b), following a relation described as the ratio of two linear expressions.^39^ This relation is also followed by the scores on PC1 (Figure 2b) plotted as a function of the XPS mole fraction of AAm (Figure 3c). The reason for this is the normalized ToF-SIMS intensities, which were used as a basis for a multivariate PCA analysis. Accordingly, the PC1 scores multiplied by the total ion intensity *I*_tot_ vary linearly with the XPS composition of the copolymer brush. In summary, the results of Figure 3 indicate that the ToF-SIMS and XPS data on the composition of copolymer brush coating correlate quite well with each other despite various effects that affect the results of both techniques to different extents,^40^ such as the matrix effect for ToF-SIMS^39^ and contamination with adventitious (oxidized) carbon for XPS.^40,41^ Also, this correlation could suggest a homogeneous with depth composition of the uppermost regions of copolymer brush coatings, taking into account different information depths of both surface sensitive techniques (<3 nm for ToF-SIMS^34^ and <10 nm for XPS^40^).

To determine the copolymer brush composition not only near the free surface of the coating but also throughout the coating depth, the dual beam profiling mode of TOF-SIMS was applied (see Figure S1 of the Supporting Information). Two samples of the P(OEGMA188-*co*-AAm)**5** brush coating (synthesized independently) were examined to determine the local mole ratio of AAm and OEGMA188 segments as a function of depth. The TOF-SIMS signals, characteristic of the AAm and OEGMA188 ion fragments and used for analysis, were carefully chosen to minimize matrix effects during ion formation (Figure S1b). The determined depth profiles reflect, as a function of the sputter time, the normalized intensity of ^30^Si^+^ ions, characteristic for glass, and the ratio of the intensities of C_3_H_3_O^+^ and C_2_H_5_O^+^ ions, reflecting the local mole ratio of AAm and OEGMA188 segments (Figure S1c,d). For both samples, a nearly constant copolymer composition is concluded through the brush coatings up to the glass substrate.

The average thickness and refractive index in the dry state of the prepared coatings were recorded using ellipsometry at room temperature, varied from 33.1 ± 1.0 to 36.9 ± 1.2 nm and from 1.493 ± 0.007 to 1.517 ± 0.001, respectively. The results of the ellipsometric measurements are presented in Table 2.

**Table 2:** Average Thickness and Refractive Index of Homopolymer and Copolymer-Grafted Brush Coatings in the Dry State at Room Temperature^a^

sample	refractive index	thickness [nm]
POEGMA188	1.509 ± 0.004	36.9 ± 1.2
P(OEGMA188-*co*-AAm)**1**	1.493 ± 0.007	33.7 ± 1.3
P(OEGMA188-*co*-AAm)**2**	1.500 ± 0.002	34.4 ± 1.1
P(OEGMA188-*co*-AAm)**3**	1.498 ± 0.002	33.6 ± 0.8
P(OEGMA188-*co*-AAm)**4**	1.495 ± 0.003	33.1 ± 1.0
P(OEGMA188-*co*-AAm)**5**	1.515 ± 0.002	34.9 ± 0.9
PAAm	1.517 ± 0.001	34.1 ± 1.4

a Numbers **n** denote the synthesized P(OEGMA188-*co*-AAm)**n** batches.

### 3.2 Temperature-Responsive Properties of POEGMA188, P(OEGMA188-*co*-AAm), and PAAm Brush Coatings Dependent on Brush Composition (CA, WLRS, AFM)

**Figure 4:**
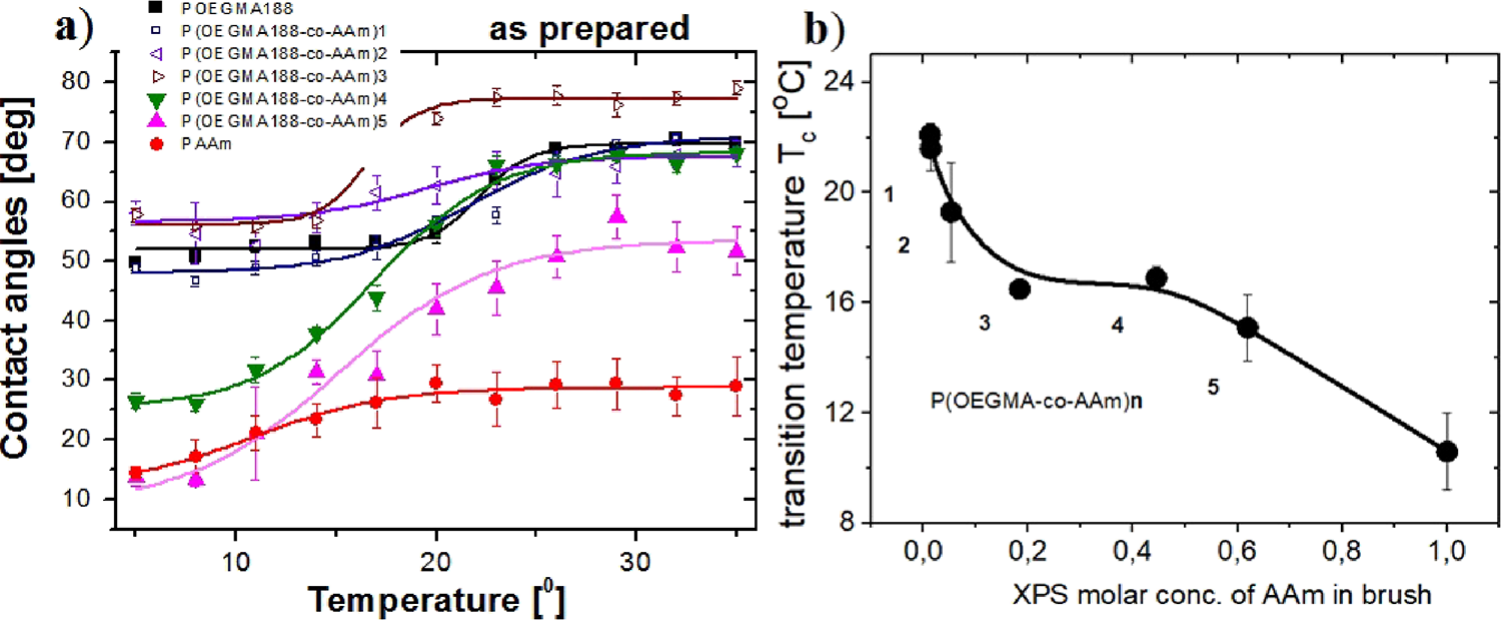
(a) Temperature dependence of water contact angles determined for brush coatings of surface grafted chains P(OEGMA188-*co*-AAm), copolymerized from di(ethylene glycol) methacrylate (OEGAM188) and acrylamide (AAm) taken with different proportions. (b) Determined transition temperatures *T*_c_ plotted versus XPS mole fraction of AAm in brush. Numbers **n** denote the synthesized P(OEGMA188-*co*-AAm)**n** batches.

To determine the temperature-responsive properties of surface grafted P(OEGMA188-*co*-AAm) brush coatings, we measured the contact angle (CA) of water droplets on coatings, determined the thickness of coatings immersed in water using white light reflectance spectroscopy (WLRS), and imaged in air coating surfaces with AFM. The contact angles of the sessile water droplets on the coatings were measured at temperatures between 5 and 36 °C. The temperature dependences of the water contact angle determined for copolymer and homopolymer brush coatings are shown in Figure 4a. Temperature-induced changes in wettability, from hydrophilic to hydrophobic, mimic the behavior typical for the lower critical solution temperature (LCST) and could be approximated by Boltzmann’s sigmoidal function that describes the contact angle *CA*(*T*) = UCA + (LCA – UCA)/(1 + exp[(*T* – *T*_c_)/slope], LCA – lower CA [°]; UCA – upper CA [°]; *T*_c_ – transition temperature [°C]; slope – rate of changes in wettability.^15^

Unexpectedly, we found temperature-responsive properties for the PAAm coatings with LCST-like behavior and transition temperature *T*_c_ = 10.6 ± 1.3 °C. Previously, hydrogels based on acrylamide and rich in AAm, with *N*,*N*′-methylene bis(acrylamide) used as a bifunctional cross-linker, were reported to exhibit LCST but at temperatures close to human body temperature.^42^ Brush coatings based on the POEGMA188 homopolymer were characterized by a sharp transition at 22.1 ± 0.4 °C, similar to our previous results.^9,43,44^ Copolymer brush coatings from a synthesis batch P(OEGMA188-*co*-AAm)**1**, with a composition almost equal to that of POEGMA188, show a similar temperature relation of wettability with slightly lower transition temperature *T*_c_. On the contrary, the copolymer brush coatings rich in OEGMA188, P(OEGMA188-*co*-AAm)**2** and P(OEGMA188-*co*-AAm)**3**, with mole fractions of AAm equal to 5.5% and 18.4%, respectively, are more hydrophobic and demonstrate reduced *T*_c_ values. In turn, copolymer brushes rich in AAm, P(OEGMA188-*co*-AAm)**4** and P(OEGMA188-*co*-AAm)**5**, with AAm mole fractions equal to 44.5% and 61.9%, respectively, demonstrate enhanced temperature-dependent response in wettability, with *T*_c_ = 15.1 ± 1.2 and 16.9 ± 0.4 °C, respectively.

The values of lower and upper contact angles (CA), their differences (Δ), and transition temperatures *T*_c_, determined for the POEGMA188, P(OEGMA188-*co*-AAm), and PAAm brush coatings (with Boltzmann’s function fitted to the CA data of Figure 4a), are summarized in Table 3. The most expressed changes in wettability are visible for copolymer coatings rich in AAm, P(OEGMA188-*co*-AAm)**4** and P(OEGMA188-*co*-AAm)**5**, where the differences between lower and upper contact angles Δ [°] are nearly 44°. These temperature-induced changes are strongly reduced for the coatings based on homopolymers and copolymers rich in OEGMA188. In addition, it might be noted that the latter, represented by P(OEGMA188-*co*-AAm)**2** and P(OEGMA188-*co*-AAm)**3**, are more hydrophobic than the POEGMA188 coatings.

**Table 3:** Lower and Upper Contact Angles (CA), Their Differences (Δ), and Transition Temperatures *T*_c_ Determined for the POEGMA188, P(OEGMA188-*Co*-AAm), and PAAm Coatings^a^

sample	lower CA [°]	upper CA [°]	Δ [°]	T_c_ [°C]
POEGMA188	52.0 ± 0.5	69.7 ± 0.7	17.7	22.1 ± 0.4
P(OEGMA188-*co*-AAm)**1**	47.9 ± 1.0	70.9 ± 1.5	23.0	21.6 ± 0.8
P(OEGMA188-*co*-AAm)**2**	56.6 ± 1.3	67.6 ± 1.0	11.0	19.3 ± 1.7
P(OEGMA188-*co*-AAm)**3**	56.1 ± 0.7	77.2 ± 0.8	21.1	16.5 ± 0.3
P(OEGMA188-*co*-AAm)**4**	25.2 ± 1.4	68.3 ± 0.8	43.1	16.9 ± 0.4
P(OEGMA188-*co*-AAm)**5**	8.8 ± 4.7	53.5 ± 3.1	44.7	15.1 ± 1.2
PAAm	11.7 ± 2.5	28.7 ± 0.6	17.0	10.6 ± 1.3

a Numbers **n** denote the synthesized P(OEGMA188-*co*-AAm)**n** batches.

Transition temperatures *T*_c_ of the brush coatings, plotted versus the XPS mole fraction of AAm in the brush, are presented in Figure 4b. Surprisingly, the observed dependence is not linear. Transition temperatures decrease sharply for the AAm mole fraction in the copolymer brush lower than 19%, remain almost constant for the AAm mole fraction ranging from 19% to ca. 50%, and again decrease for even higher AAm concentrations.

**Figure 5:**
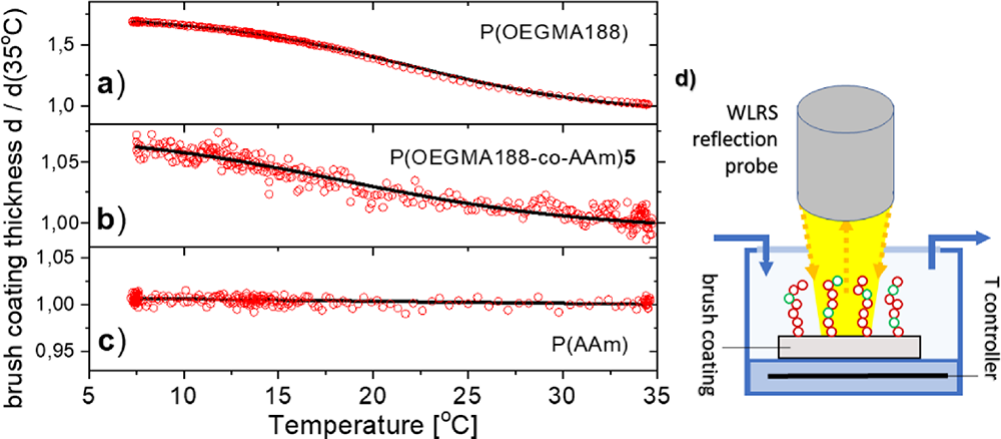
Temperature dependence of the relative thickness of the brush coating with respect to that at 35 °C, determined with WLRS *in situ* for the samples immersed in water: (a) POEGMA188, (b) P(OEGMA188-*co*-AAm)**5**, and (c) PAAm. Each point represents the average of measurements of at least three different spots of each sample, performed at a temperature range between 5 and 35 °C. (d) Schematic of the applied WLSR configuration.

To analyze *in situ* in water the temperature response of the synthesized brush coatings, we applied a WLRS platform (see Figure 5d)^35,36^ for the first time, examining with a fiber optic probe the spectrum of white light reflected from a brush coating placed in a liquid cell with controlled temperature. The results of such measurements, performed between 5 and 35 °C for the POEGMA188 and PAAm homopolymers and the P(OEGMA188-*co*-AAm)**5** copolymer coatings, are presented in Figure 5a,b,c. The relative thickness of the brush coatings with respect to that at 35 °C is plotted as a function of temperature, and each point represents the average of measurements in at least three different spots in each sample. As expected, the POEGMA188 brush coating demonstrated a well-expressed transition at 21.8 ± 0.1 °C (Figure 5a). The same LCST-like transition value was determined from the water contact angles (22.1 ± 0.4 °C). The ratio of coating thickness at the swollen and collapsed state is equal to 1.67 and is similar to the results reported earlier.^9,45^ In turn, unexpectedly, the PAAm coating does not show any detectable changes in thicknesses upon temperature variation. Here, the coating thickness ratio of the more hydrophilic and more hydrophobic state is close to 1. Finally, the P(OEGMA188-*co*-AAm)**5** coating shows a slight temperature response of thickness with the thickness ratio of the states below and above *T*_c_ equal to ∼1.07. The value of the transition temperature *T*_c_ determined with WLRS is slightly higher (18.4 ± 0.5 °C) than that determined based on wetting contact angles (15.1 ± 1.2 °C). The comparison of contact angle measurements and WLRS results obtained suggest different mechanisms of the temperature response for different brush coatings, with a sharply expressed ‘classical’ transition between hydrated loose coils and hydrophobic collapsed chains for POEGMA188, and changes in hydrophilic–hydrophobic balance without collapsed chains for P(OEGMA188-*co*-AAm)**5** and PAAm.

**Figure 6:**
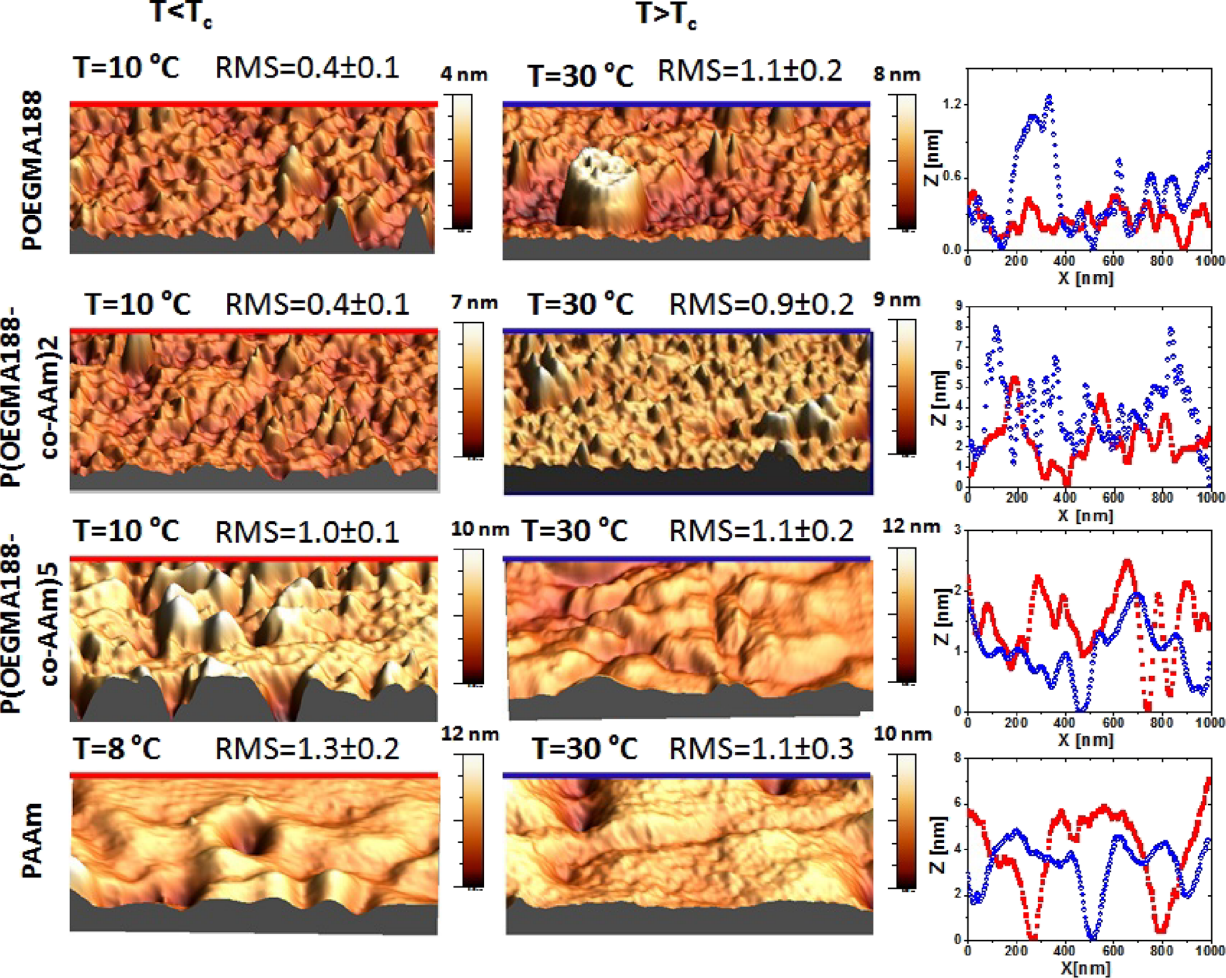
AFM images and cross sections of the as-prepared POEGMA188, P(OEGMA188-*co*-AAm)**2**, P(OEGMA188-*co*-AAm)**5**, and PAAm brush coatings recorded at temperatures below and above *T*_c_. The RMS values (in nanometers) characterize the morphology.

**Figure 7:**
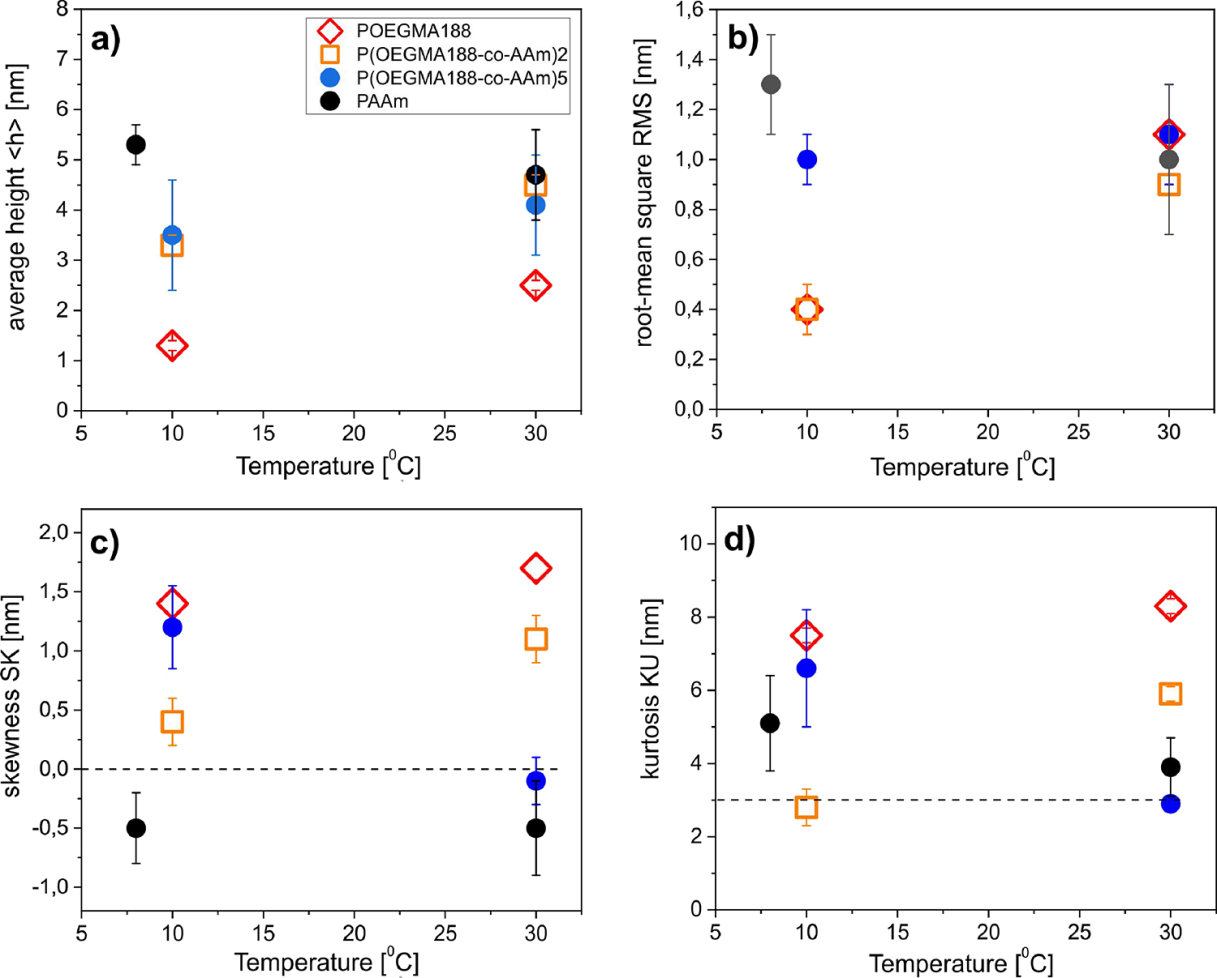
Parameters of the height distribution of topographic AFM data, recorded below and above *T*_c_ for the as-prepared POEGMA188, P(OEGMA188-*co*-AAm)**2**, P(OEGMA188-*co*-AAm)**5**, and PAAm brush coatings: (a) average height ⟨h⟩, (b) root-mean square RMS, (c) skewness SK, and (d) kurtosis KU.

The surfaces of the as-prepared brush coatings were imaged in air with AFM at 10 and 30 °C for POEGMA188, P(OEGMA188-*co*-AAm)**2**, and P(OEGMA188-*co*-AAm)**5** and at 8 and 30 °C for PAAm. The representative AFM results obtained are shown in Figure 6. Furthermore, the height distribution parameters of the topographic AFM data are presented in Figure 7. These parameters reflect different moments of height distribution, i.e., its mean value (average height ⟨h⟩), spread (root-mean square RMS), asymmetry (skewness SK), and tailedness (kurtosis KU). We start the topographic analysis noting that all surfaces exhibit kurtosis KU close to or higher than 3 (Figure 7d), reflecting the existence of inordinately high peaks or deep valleys (Figure 6). In turn, the positive and negative SK skewness values indicate surfaces dominated by peaks and valleys, respectively (Figure 7c). In fact, at both temperatures, *T* < *T*_c_ and T < *T*_c_, peaks (SK > 0) dominate the topography of POEGMA188 and P(OEGMA188-*co*-AAm)2 and valleys (SK > 0) prevail in the case of PAAm (Figure 6). In contrast, the dominant surface features are exchanged from peaks (SK > 0) below *T*_c_ to valleys (SK slightly below 0) above *T*_c_ for grafted brush coatings P(OEGMA188-*co*-AAm)**5** (Figure 6). Finally, an exceptionally low roughness value (RMS ∼ 0.4 nm, Figure 7b) indicates a smooth morphology of POEGMA188 and P(OEGMA188-*co*-AAm)**2** recorded at *T* < *T*_c_ (Figure 6). Both morphologies are similar, but the vertical extent is larger for P(OEGMA188-*co*-AAm)**2**, as indicated by a higher value of average height ⟨h⟩ (Figure 7a). Also, both POEGMA188 and P(OEGMA188-*co*-AAm)**2** brush coatings exhibit a temperature-induced transition from smooth to rough surface topography, reflected by RMS values (Figure 7b), somewhat less pronounced for P(OEGMA188-*co*-AAm)**2**. In striking contrast, neither PAAm nor the P(OEGMA188-*co*-AAm)**5** copolymer shows such a transition with essential changes in the RMS values at *T*_c_, suggesting that the thermal response mechanisms are different from those postulated for POEGMA188. Comparable RMS values at *T* < *T*_c_ and *T* > *T*_c_ suggest weak dehydration that might modify the morphology of the coatings.

**Figure 8:**
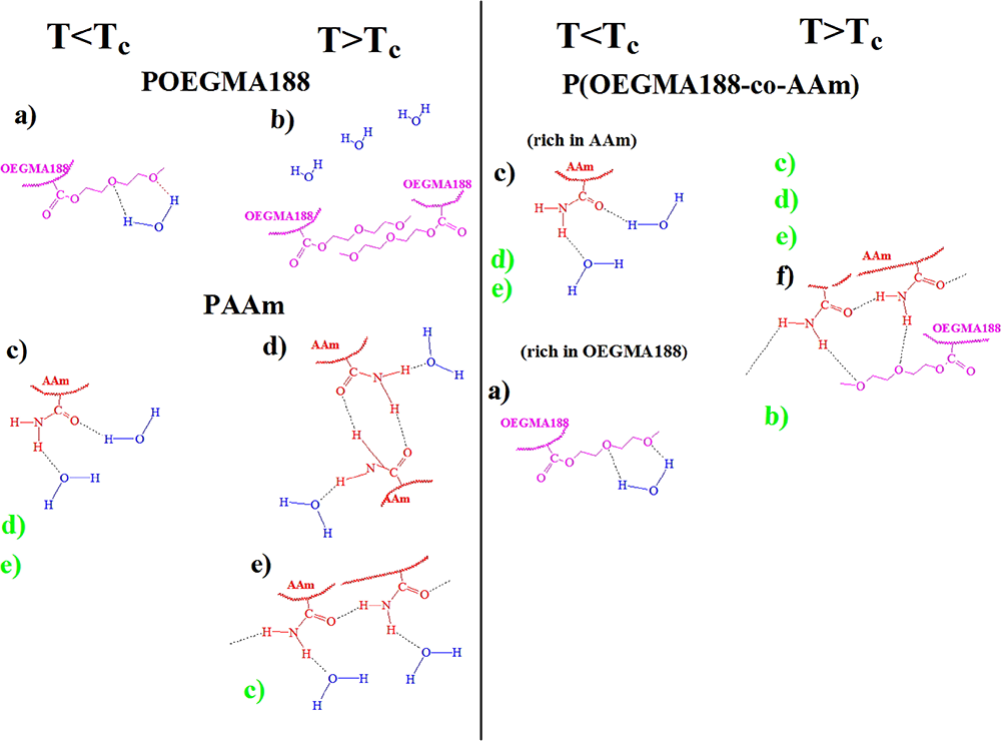
Hypothetical conformations of hydrogen bonding between water, amide groups, and ether groups for POEGMA188 (a, b), PAAm (c, d, e), and P(OEGMA188-*co*-AAm) brush coatings (a, b, c, d, f) above (left columns) and below transition temperature *T*_c_ (right columns). The letters in black mark dominant conformations and those in green are minor conformations at different polymer states.

**Figure 9:**
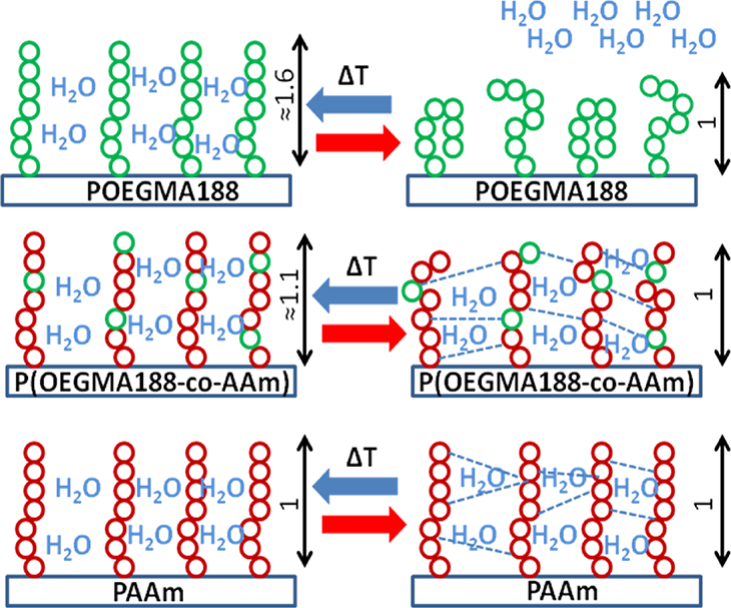
Hypothetical behavior of POEGMA188, P(OEGMA188-*co*-AAm), and PAAm brush coatings at *T* < *T*_c_ and *T* > *T*_c_ . Dashed lines mark hydrogen bonds between segments of AAm (brown) and OEGMA188 (green), dominant above *T*_c_, which do not destroy (as van der Waals interactions for POEGMA188) but only change the arrangement of hydrogen bonds between the segments and water molecules. As a result, a minimal thermal response of coating thickness or morphology coexists with a distinct change in wettability.

The behavior of POEGMA brush coatings is well known and described in numerous works.^9,43−45^ The typical thermal response of wettability and morphology with well-expressed LCST is mainly attributed to the hydrogen bonds between the ether oxygens of poly(ethylene glycol) and water hydrogens at *T* < *T*_c_ (see Figure 8, structure a). This balance is disrupted at temperatures elevated above room temperature *T* > *T*_c_, where polymer–polymer interactions are thermodynamically favored compared to polymer–water interactions (Figure 8, structure b). A sharp transition from the swollen state to the collapsed state resulting in changes in the hydrophobicity of the POEGMA188 coatings and their thickness is depicted in Figure 9.

In turn, PAAm in water at room temperature (that is, above *T*_c_) forms different hydrogen bond structures,^46−51^ including free amide groups (Figure 8, structure c), *cis-trans*-multimers (Figure 8, structure d), and *trans*-multimers (Figure 8, structure e) of amide groups. However, the amount of *cis-trans*-associates as well as *trans*-associates of the amide groups in PAAm is significantly smaller than that of free amide groups. Based on experimental results (Figures 4a, 5c, 6, and 7) and previous studies, we postulate the mechanism of thermal response for PAAm, which differs from the ‘classical’ transitions based on LCST shown earlier for P*N*IPAM, POEGMA, and many other polymers.^9^ We expect that this behavior is mainly attributed to conformations of the hydrogen bonds between hydrophilic amide groups of PAAm segments and water (Figure 8, structure c), which are dominant at lower temperatures (as in the case of the P*N*IPAM and similar polymers) but are slightly changed by the hydrogen bonds among the amide groups in the PAAm chains (Figure 8, structures d and e) above the transition temperature, mimicking LCST. Most probably, temperature-induced dehydration of free amide groups (Figure 8, structure c) can lead to the formation of hydrogen bonds between amide fragments (Figure 8, structures d and e), which can still be hydrated. This is in contrast to P*N*IPAM, where hydrogen bonds with water are almost completely replaced at LCST by hydrogen bonds between the amide groups in the P*N*IPAM chains.^52^ For PAAm, the ratio of free amide groups that form hydrogen bonds with water and amide groups that form such bonds between themselves is thermodynamically balanced and changes only slightly at transition, while the total number of water molecules bonded with amide groups is not much modified. Note an essential impact of the transition on the wettability (see Figure 4a) but almost no effect on the morphology (see Figure 6) and thickness of the PAAm coating (Figure 5c). This suggests that water molecules water are stored in PAAm-grafted brushes and that a temperature-induced change in wettability is realized only by the change of conformations of hydrogen bonds in polymer coating, where water molecules are caged (see Figure 9).

In contrast to relatively simple mechanisms of transition for PAAm and POEGMA188, we expect that the mechanism of transition for copolymer P(OEGMA188-*co*-AAm) is very complicated, including hydrogen bonding among the ether groups of OEGMA188 and the amide groups of AAm, as well as competing interactions between the copolymer segments and water. This mechanism depends on the ratio of the mers in the copolymer composition. Previously, Zheltonozhskaya et al.^46−48,53,54^ demonstrated the participation of the transmultimers of the amide groups of triblock copolymers in the formation of the hydrogen bond system between the poly(ethylene oxide) and PAAm segments (see Figure 8, structure f). The enhanced temperature-dependent response of wettability for P(OEGMA188-*co*-AAm) copolymers with a high mole fraction of hydrophilic AAm (see Figure 4a), not accompanied by any significant changes in coating thickness (see Figure 5), suggests the formation at transition temperature *T*_c_ of more hydrophobic structures realized by hydrogen bonding between ether oxygens of OEGMA188 and amide fragments of AAm (see Figure 8, structure f) where water molecules are caged. We believe that the amount of dehydrated free amide groups is relatively low at a temperature below *T*_c_ and an elevated temperature stimulates their dehydration and the formation of new hydrogen bonds between AAm and OEGMA188 units (Figure 8, structure f). Also, this hypothesis explains why the P(OEGMA188-*co*-AAm) coatings with a high mole fraction of OEGMA188 are at *T* < *T*_c_ more hydrophobic than “pure” POEGMA188 brushes (see Figure 4a). In this case, the majority of amide groups form hydrogen bonds with ether groups of OEGMA188 and a temperature-induced transition can be realized only by dehydration of OEGMA188, which still forms hydrogen bonds with water. A similar effect, including an increase in the length of hydrophobic hydrogen-bonded parts between poly(ethylene oxide) and PAAm blocks with growth of poly(ethylene oxide), was observed for triblock copolymers of poly(ethylene oxide) and PAAm.^53,54^

### 3.3 Temperature-Responsive Properties of the Brush Coatings Immersed in pH Buffer Solutions (CA, AFM): ‘Schizophrenic’ Behavior of P(OEGMA188-*co*-AAm)**5**

**Figure 10:**
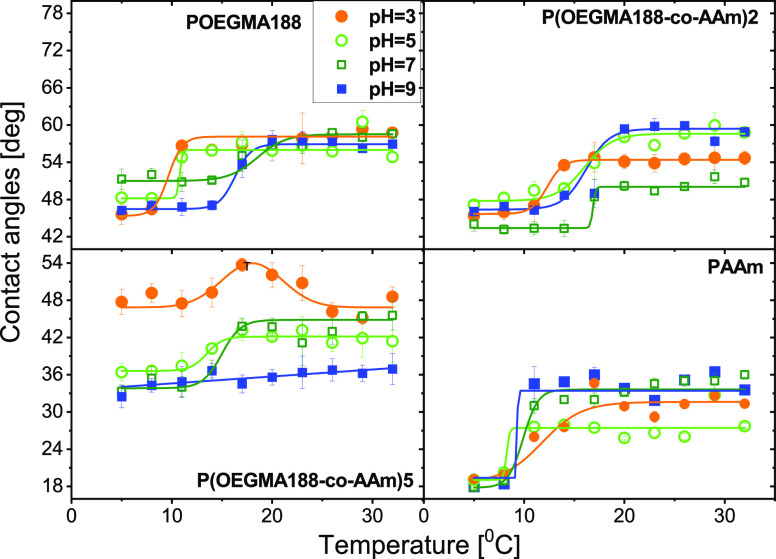
Temperature dependence of water contact angles determined for brush coatings of surface grafted chains of homopolymers, POEGMA188 and PAAm, and copolymers P(OEGMA188-*co*-AAm) rich in OEGMA188 and AAm, after immersion in pH buffer solution with various pH values. The numbers **n** denote the synthesized batches of P(OEGMA188-*co*-AAm)**n**.

pH buffer solutions are well known to have a strong impact on protein stability and cell viability. Therefore, the behavior of brush coatings in buffer solutions at different temperatures plays a key role in their applications in biomedicine. The temperature dependence of water contact angles determined for homopolymer, POEGMA188 and PAAm, and copolymer brush coatings rich in OEGMA188 and AAm, P(OEGMA188-*co*-AAm)**2** and P(OEGMA188-*co*-AAm)**5**, after their immersion in the buffers with various pH (pH = 3, 5, and 7 for phosphate–citrate buffer and pH = 9 for borate buffer) is presented in Figure 10 and Table 4. Contact angle measurements were performed at a temperature ranging from 5 to 32 °C for the coatings, as prepared and after their incubation in a pH buffer solution with pH equal to 3, 5, 7, and 9. In general, the wettability change around the transition is lower for the coatings after immersion in a buffer solution than that for the as-prepared coatings.

**Table 4:** Lower and Upper Contact Angles (CA), Their Differences (Δ), and Transition Temperatures *T*_c_ Determined for POEGMA188, P(OEGMA188-*Co*-AAm), and PAAm after Immersion in Various pH Buffer Solutions^a^

sample	buffer solution	lower CA [°]	upper CA [°]	Δ [°]	*T*_c_ [°C]
POEGMA188	water	52.0 ± 0.5	69.7 ± 0.7	17.7	22.1 ± 0.4
	pH = 3	45.4 ± 2.7	58.1 ± 0.5	12.7	9.6 ± 1.0
	pH = 5	48.2 ± 2.2	56.0 ± 0.4	7.8	10.7 ± 1.0
	pH = 7	51.0 ± 0.6	58.5 ± 0.3	7.5	17.5 ± 0.9
	pH = 9	46.5 ± 0.3	56.9 ± 0.7	10.4	16.3 ± 0.5
P(OEGMA188-*co*-AAm)**2**	water	56.6 ± 1.3	67.6 ± 1.0	11.0	19.3 ± 1.7
	pH = 3	45.7 ± 0.3	54.4 ± 0.2	8.7	12.3 ± 0.3
	pH = 5	47.8 ± 0.4	58.6 ± 0.4	10.8	16.0 ± 0.6
	pH = 7	43.4 ± 0.5	50.1 ± 0.3	6.7	16.9 ± 0.7
	pH = 9	46.4 ± 0.5	59.4 ± 0.5	13	16.2 ± 0.9
P(OEGMA188-*co*-AAm)**5**	water	8.8 ± 4.7	53.5 ± 3.1	44.7	15.1 ± 1.2
	pH = 3	LCST-like at 14.2 °C and UCST-like at 23.0 °C			
	pH = 5	36.6 ± 0.3	42.1 ± 0.2	5.5	13.5 ± 0.7
	pH = 7	33.8 ± 0.7	44.8 ± 0.5	11	15.0 ± 0.6
	pH = 9	no temperature-induced transition			
PAAm	water	11.7 ± 2.5	28.7 ± 0.6	17.0	10.6 ± 1.3
	pH = 3	18.5 ± 1.7	31.6 ± 0.6	13.1	11.8 ± 1.2
	pH = 5	19.1 ± 2.8	27.4 ± 0.7	8.3	8.2 ± 2.5
	pH = 7	17.7 ± 0.6	33.6 ± 0.5	15.9	9.9 ± 0.6
	pH = 9	19.4 ± 1.1	33.4 ± 0.7	14	9.3 ± 0.7

a The numbers **n** denote the synthesized batches of P(OEGMA-*co*-AAm)**n**.

The impact of various pH buffer solutions on the temperature-responsive properties of the POEGMA188 brush coatings was described in detail in our previous work.^55^ In all cases, the incubation of POEGMA188 coatings in buffer solutions induces a shift of the *T*_c_ point from 22 °C, determined for the prepared samples, to 9–11 °C and 16–17 °C for acid and neutral/base buffer, respectively (see Figure 10 and Table 4). Completely different behavior was recorded for PAAm coatings, where transition temperature *T*_c_ is equal to 10 °C and no essential impact of the various buffers on *T*_c_ is observed (Figure 10 and Table 4). Analysis of the temperature relations obtained for the P(OEGMA188-*co*-AAm)**2** copolymer coating rich in OEGMA188 mers reveals the reduction of *T*_c_ values after immersion of the coating in all buffer solutions, with the strongest impact observed after coating incubation in phosphate–citrate buffers with pH equal to 3 (Figure 10 and Table 4).

Special attention should be paid to the copolymer brush coating with a high content of AAm mers, represented by P(OEGMA188-*co*-AAm)**5**. For these samples, immersion in pH buffer solutions leads to a ‘schizophrenic’ behavior in wettability (see Figure 10 and Table 4), not reported so far for surface-grafted brush coatings and dependent on buffer pH: For pH = 3, the shape of the temperature dependence of the water contact angle (Figure 10) indicates a transition from hydrophilic to hydrophobic at *T*_c_ = 14.2 °C that resembles LCST followed by a reversed change (from hydrophobic to hydrophilic) at *T*_c_ = 23.0 °C, which mimics the behavior typical for the upper critical solution temperature (UCST). In turn, for pH = 5 and 7, only the transition that resembles LCST is sustained, but the change in wettability around the transition, expressed by the difference Δ between the upper and lower CA, is strongly reduced compared to immersion in ‘pure’ water (Δ = 6 and 11°, respectively, vs 45°). Finally, for pH = 9, the temperature-induced transitions in wettability are blocked.

**Figure 11:**
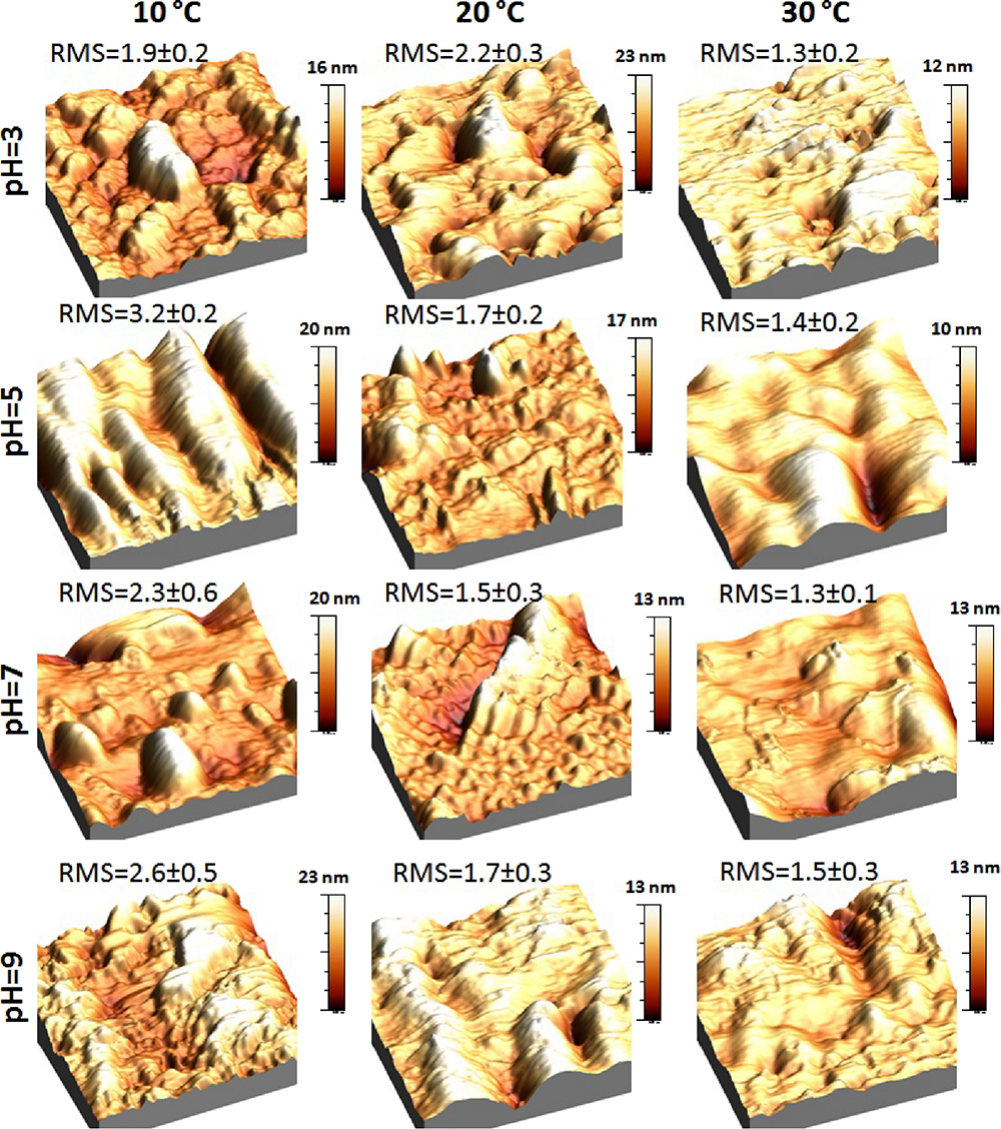
Topography of the P(OEGMA188-*co*-AAm)**5** coating, recorded with AFM at 10, 20, and 30 °C (columns) after immersion of the samples in buffer solution with pH equal to 3, 5, 7, and 9 (rows). The RMS values (in nanometers) characterize the morphology in each case.

To characterize more completely the P(OEGMA188-*co*-AAm)**5** coatings, which are the most interesting and prospective for biomedical applications, AFM images were recorded in air at 10, 20, and 30 °C after immersion in buffer solutions with pH equal to 3, 5, 7, and 9. The results obtained are presented in Figure 11. In contrast to their as prepared counterparts, where the comparable RMS values are determined for 10 and 30 °C, here, the RMS values are higher for 10 °C than for 30 °C. Although AFM images recorded for pH 3, 5, 7, and 9 were obtained for different types of pH buffer solutions (phosphate and borate buffers), the recorded topographies at 10 °C (*T* < *T*_c_) are very similar. They present relatively high island-shaped structures, elongated for pH = 5. In turn, AFM images obtained for pH 3, 5, and 7 at 20 and 30 °C (*T* > *T*_c_) demonstrate significantly narrower and lower structures. Surprisingly, although P(OEGMA188-*co*-AAm)**5** after immersion in a buffer solution with pH = 9 does not exhibit changes in wettability for all temperatures tested, it shows well-expressed changes in coating morphology, similar to the ones recorded for coatings after immersion in buffer solutions with pH equal to 5 and 7 where the temperature-induced transition was expressed by changes in wettability.

The pH-modulated temperature-induced changes in the coating wettability and morphology are essentially different from those in pure water and suggest more complicated mechanisms of transition. It is likely that the ions of the buffer salt play no last role in the regulation of the coating behavior. Blocking of the transition at pH = 9 may be related to hydrolysis of the amide groups in base buffers, as was described in the work,^56^ where a high hydrolysis rate was observed under alkaline conditions of the acrylamide mers. The resulting carboxylic groups are in a nonionized state and interaction between them and the ether groups of POEGMA188 is impossible. The existence of the transition for pH = 3, at *T*_c_ = 14.2 °C, that resembles LCST followed by a reversed transition at *T*_c_ = 23.0 °C, which mimics the behavior typical for UCST, is not expected and cannot be explained precisely at this moment. Most likely, additional hydrogen bonds are created using various buffer components.

## 4 Summary and Conclusions

In the presented work, we synthesized a series of novel temperature-responsive copolymer brush coatings with P(OEGMA188-*co*-AAm) chains grafted from glass surfaces functionalized with (3-aminopropyl)triethoxysilane and then with the ATRP initiator. Synthesis of the brush coatings was confirmed by XPS and ToF-SIMS.

The temperature-responsive properties of POEGMA188 brush coatings are well known and are attributed to hydrogen bonds between the ether oxygens of poly(ethylene glycol) and water hydrogens at *T* < *T*_c_ and their disruption at *T* > *T*_c_. Unexpectedly, PAAm coatings also present temperature-responsive properties with transition temperature *T*_c_ = 10.6 ± 1.3 °C, probably realized by partial dehydration of free amide groups and the formation of hydrogen bond structures such as *cis-trans*-multimers and *trans*-multimers of amide groups. The combination of OEGMA188 and AAm units in grafted copolymer brushes with a high mole fraction of AAm (>44%) dramatically improves the response in wettability around *T*_c_ (with the change in the water contact angle increased from 17–18 to 45°) but this thermal response is hardly manifested by changes in the thickness and morphology of the coating. This suggests the formation at *T*_c_ of more hydrophobic structures realized by hydrogen bonding between ether oxygens from OEGMA188 and amide fragments of AAm, which changes but does not destroy the arrangement of hydrogen bonds between mers and water molecules. This mechanism is enabled by the copolymer composition being homogeneous with depth in the brush coatings.

Furthermore, P(OEGMA188-*co*-AAm) coatings with a high mole fraction of AAm demonstrate ‘schizophrenic’ behavior in wettability after immersion in pH buffer solutions, with transitions that mimic LCST and UCST for pH = 3, LCST for pH = 5 and 7, and temperature-induced transitions blocked for pH = 9.

The resulting temperature-responsive grafted brush coatings can be used in at least five advanced biomedical applications: (1) bacteria killing and (2) biologically active substance release, (3) temperature-controlled protein adsorption, (4) high viability cell harvesting, and (5) temperature-controlled cell and tissue detachment.^57^ Despite advancements in the creation of such fascinating materials, a number of problems still need to be resolved, including biocompatibility, high efficacy, selectivity of action, stability, long-term and multiple uses, and the temperature of the transition being close to physiological temperatures (appropriate transition temperature). As a result, fresh ideas are constantly needed to create surfaces that might satisfy all necessary criteria. Compared to current analogs, this type of grafted brush coating provides at least three benefits. First, wettability variations caused by temperature are well exhibited and considerably better than for POEGMA188 and PAAm. Second, the end products exhibit simultaneous thermo- and pH-responsiveness. Third, they exhibit ″schizophrenic″ behavior, thereby expanding the range of potential regimes for their uses.

## Supporting Information Available

Scheme of synthesis of P(OEGMA188-*co-*AAm) brush coatings on the glass surface, consisting of glass silanization with APTES, grafting of the ATRP initiator, and copolymerization using SI-ATRP; results of the surface spectroscopy mode of TOF-SIMS, used to choose the signals characteristic for the AAm and OEGMA188, and results of the dual beam profiling mode of TOF-SIMS, reflecting the local mole ratio of AAm and OEGMA as a function of depth in the copolymer brush coating (PDF)
