# Supplementary material for: Temperature- and pH-Responsive Schizophrenic Copolymer Brush Coatings with Enhanced Temperature Response in Pure Water
Source: ACS Appl Mater Interfaces. 2023 Feb 3;15(6):8676–90. doi: 10.1021/acsami.2c20395 (PMC9940115; doi:10.1021/acsami.2c20395)
Supplement: Supplementary file 1 — am2c20395_si_001.pdf [file am2c20395_si_001.pdf]

**Supporting Information**

# Temperature- and pH-Responsive Schizophrenic Copolymer Brush Coatings with Enhanced Temperature-Response in Pure Water

*Yana Shymborska[1,2], Yurij Stetsyshyn[1,2*], Kamil Awsiuk[1], Joanna Raczkowska[1], Andrzej Bernasik[3], Natalia Janiszewska[1], Pawel Dąbczyński[1], Andrij Kostruba[4], Andrzej Budkowski[1*]*

[1]Smoluchowski Institute of Physics, Jagiellonian University, Łojasiewicza 11, 30-348 Kraków, Poland

[2]Lviv Polytechnic National University, St. George's Square 2, 79013 Lviv, Ukraine

[3]Faculty of Physics and Applied Computer Science, AGH - University of Science and Technology, al. Mickiewicza 30, 30-049 Kraków, Poland

[4] Faculty of Food Technologies and Biotechnology, Stepan Gzhytskyi National University of Veterinary Medicine and Biotechnologies of Lviv, Pekarska 50, 79000, Lviv, Ukraine

[*]Corresponding authors E-mail: Yurij Stetsyshyn yrstecushun@ukr.net; Andrzej Budkowski andrzej.budkowski@uj.edu.pl

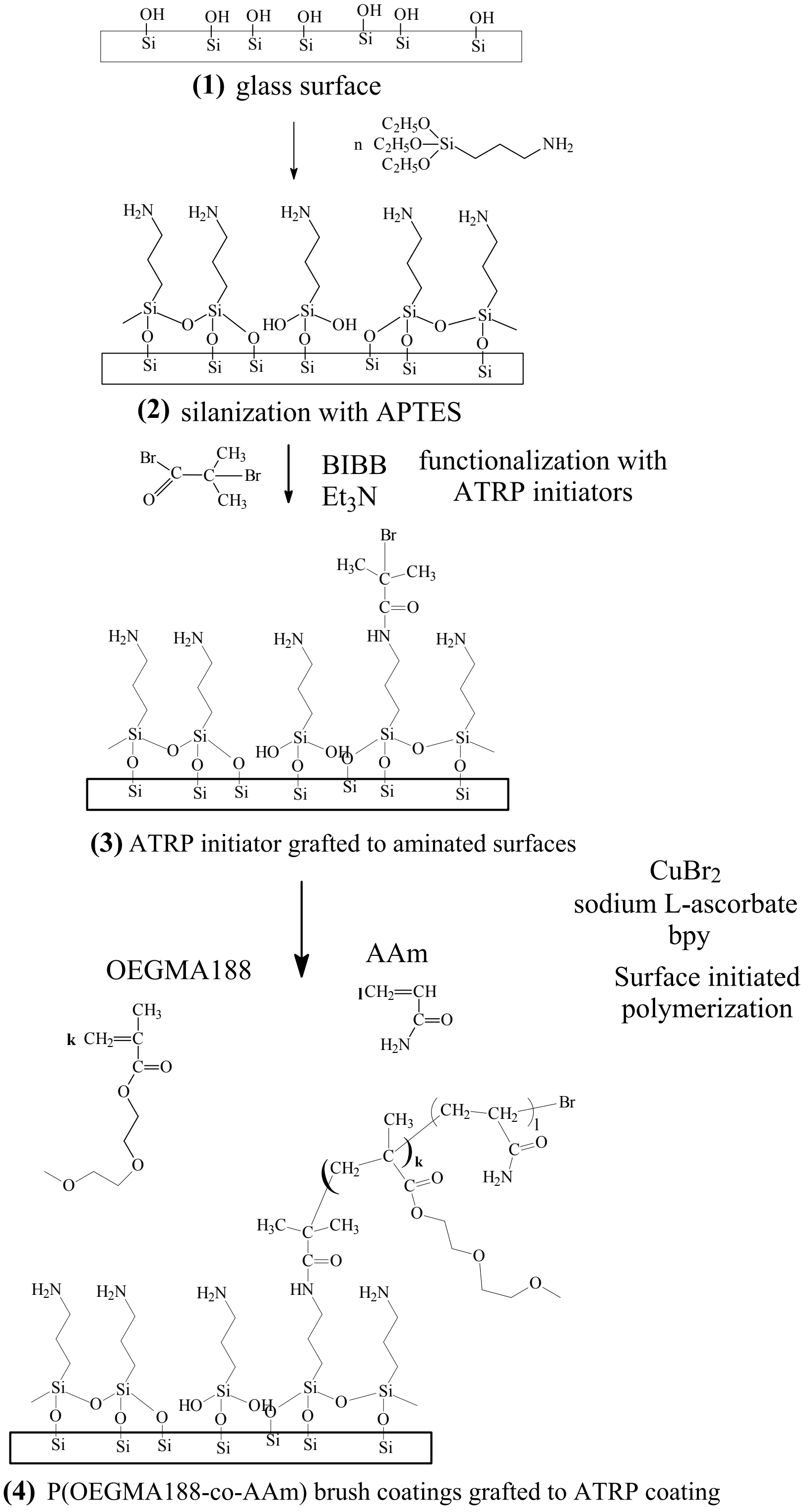


**Scheme S1.** Functionalization of glass surface **(1)** with amino-terminated APTES film **(2),** subsequent grafting of ATRP initiator **(3)** and polymerization of OEGMA188 and AAm, initiated by ATRP initiator, resulting in P(OEGMA188-*co*-AAm) **(4)** copolymer brush coatings.

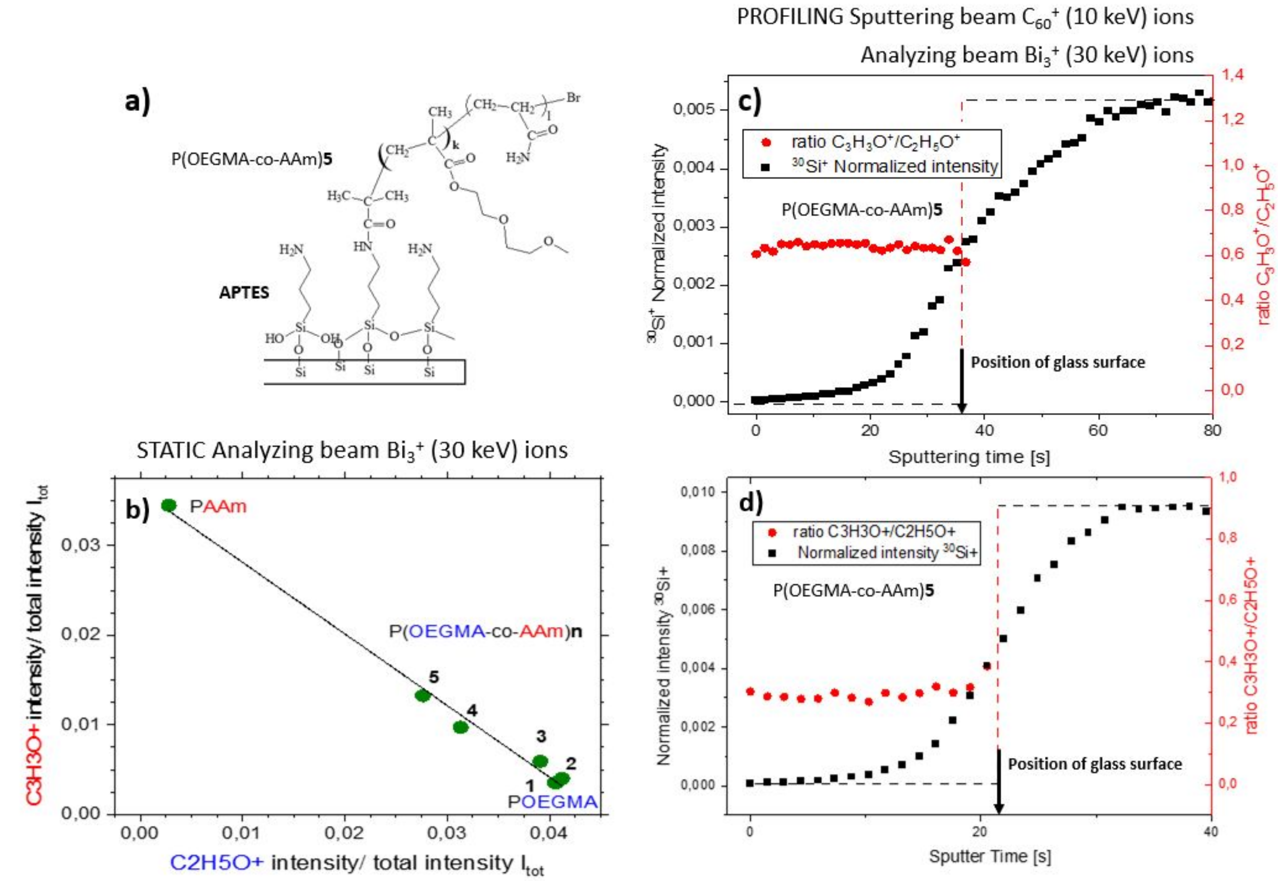


**Figure S1.** P(OEGMA188-*co*-AAm) copolymer brush coatings grafted to a glass surface functionalized with APTES (**a**) and the results of TOF-SIMS surface spectroscopy (**b**), which applies a $Bi_3^+$ (30 keV) ion beam, and the dual beam depth profiling mode of TOF-SIMS (**c, d**) using the $C_{60}$+ (10 keV) ion beam for sputtering and the $Bi_3^+$ (30 keV) ion beam for analysis. (**b**) Inverse correlation between the normalized intensities of TOF-SIMS signals characteristic for AAm ($C_3H_3O^+$) and OEGMA ($C_2H_5O^+$), plotted for PAAm, POEGMA and various batches **n** of P(OEGMA-*co*-AAm)**n** brushes. Since the normalized intensity of the $C_3H_3O^+$ signal correlates with the XPS composition of the copolymer brush (see the main text), this indicates a negligible matrix effect on the formation of both the $C_3H_3O^+$ and $C_2H_5O^+$ ions chosen for subsequent analysis with dual beam depth profiling. (**c, d**) TOF-SIMS depth profiles determined for two different samples (synthesized independently) of the brush coating P(OEGMA-*co*-AAm)**5**, represented as a function of the sputter time for the normalized intensity of $^{30}Si^+$ ions, characteristic for glass, and the ratio of the intensities of $C_3H_3O^+$ and $C_2H_5O^+$ ions, reflecting the local mole ratio of AAm and OEGMA segments in the copolymer brush coating. The inflection point of the $^{30}Si^+$ signal marks the position of the glass surface, and the depth profile reflecting organic ion fragments is not considered for longer sputtering times.
